# Tricuspid Valve Infective Endocarditis in People Who Inject Drugs: A Single-Center Retrospective Observational Study of Percutaneous Mechanical Aspiration and a Surgical Approach

**DOI:** 10.3390/idr18050092

**Published:** 2026-08-24

**Authors:** Lauren Bernard, Juliana S. Sherchan, Nazary Nebeluk, David Zapata, Murtaza Dawood, Douglas Anderson, Ramon A. Riojas, Shivakumar Narayanan

**Affiliations:** 1 Department of Internal Medicine, University of Maryland School of Medicine, Baltimore, MD 21201, USA; 2 Division of Clinical Care and Research, Institute of Human Virology, University of Maryland School of Medicine, Baltimore, MD 21201, USA; 3 Division of Cardiac Surgery, University of Maryland School of Medicine, Baltimore, MD 21201, USArriojas@som.umaryland.edu (R.A.R.); 4 Luminis Health Anne Arundel Medical Center, Annapolis, MD 21401, USA

**Keywords:** infective endocarditis, injection drug use, substance use, opioid use disorder, percutaneous mechanical aspiration

## Abstract

Background: People who inject drugs (PWID) comprise the majority of patients with native tricuspid valve (TV) infective endocarditis (IE). Many patients require surgical repair or replacement of their TV, but some may be deemed ineligible for surgical management due to high operative risk or patient preference. Vacuum-assisted percutaneous mechanical aspiration (PMA) has emerged as a potential alternative to surgical management in this population. The objective of this study was to describe real-world patient characteristics and clinical outcomes in PWID who underwent PMA or surgical management of TV IE. Methods: We retrospectively reviewed PWID hospitalized with endocarditis at a single tertiary care center (2016–2025) who underwent an isolated PMA or surgical TV repair/replacement (TVR). Baseline demographic, clinical, microbiologic, and echocardiographic characteristics were described. Clinical outcomes were compared descriptively between groups, recognizing imbalances in treatment selection. Results: A total of 40 PWID met inclusion criteria; 17 underwent PMA and 23 underwent surgical TVR. There was substantial baseline clinical heterogeneity between the two groups. Procedural success was numerically higher with surgery than with PMA (95.7% vs. 88.2%). Clinical success, a composite of procedural success, treatment completion, and lack of need for reintervention, was numerically higher in the TVR group (73.9% vs. 52.9%). One-year mortality was similar between groups. Conclusions: In this single-center retrospective observational cohort of PWID with isolated TV IE, there was no statistical difference in composite clinical or procedural success between patients who underwent PMA or TVR. PMA may be a feasible source-control strategy for select patients not eligible for immediate surgery; however, larger prospective studies are needed to define optimal patient selection factors and comparative effectiveness.

## 1. Introduction

The opioid crisis in the US has been marked by a significant rise in invasive infections, including infective endocarditis (IE), with a corresponding rise in healthcare utilization related to their management [1,2]. Among people who inject drugs (PWID), IE is a serious complication and commonly involves the tricuspid valve (TV) [3,4]. PWID with IE are typically younger, by about a decade, experience more homelessness and significant poverty, and have higher rates of hepatitis C virus infection compared to people with IE who are not IV drug users (IVDU) [5]. In the US, hospitalizations among PWID with IE have increased 12-fold from 2007 to 2017 [2]. In the Italian Registry of IE, PWID accounted for 61 of 677 cases (9%), including 26 cases with right-sided IE, which generally has a better prognosis and lower rate of surgical intervention than left-sided IE [6].

Management of IE in PWID is generally consistent with the management of IE in non-IVDU, including a combination of medical and surgical management [5]. Medical management typically involves two to six weeks of parenteral antimicrobials. Surgical management typically involves open valve replacement or repair, and the decision to undergo valve surgery is individualized. Surgery prior to the completion of antibiotics is indicated for anatomic and structural concerns, such as new valvular regurgitation, persistent bacteremia, ongoing embolic phenomena, large vegetations, or the presence of high-risk organisms, such as methicillin-resistant *Staphylococcus aureus* (MRSA). Studies have noted the limited rationale for withholding surgery in PWID, with one study finding that surgical intervention for first-episode IVDU-IE was associated with improved all-cause mortality [7]. However, surgical management is still associated with higher rates of adverse clinical outcomes, including recurrent IE, reintervention, and mortality. These risks are especially pronounced in the setting of untreated substance use disorder (SUD) [2,8,9]. Vacuum-assisted percutaneous mechanical aspiration (PMA), which enables percutaneous debulking of valvular vegetations, has emerged as a potential nonoperative strategy for source control. Initially approved by the U.S. Food and Drug Administration in 2014 for intravascular thrombus removal, PMA has since been utilized for aspiration of right atrial thrombi, with growing interest in its role in managing TV IE. PMA involves inserting an aspiration catheter via the right internal jugular vein or one of the femoral veins, and a return cannula via the left femoral vein, enabling extracorporeal suction-assisted removal of intracardiac masses under real-time transesophageal echocardiographic guidance [10].

PMA is frequently utilized in patients considered poor surgical candidates due to infection severity, comorbidities, or social factors, creating important baseline differences from patients selected for surgery. Emerging data suggest that PMA may offer favorable short-term outcomes in selected patients. One systematic review of 44 studies, including 301 patients, reported clinical success in 79.1% and a survival rate of 89.7% in patients undergoing PMA [11]. A single-center study reported a 60–100% decrease in vegetation size with a 100% clearance of bacteremia post PMA [12]. In a retrospective cohort study, El-Dalati et al. found no difference in 30-day and one-year mortality of patients who underwent surgical intervention compared with PMA [13]. While patient outcomes after PMA have generally been favorable, more evidence is needed to clarify the indications for using PMA compared to surgical intervention. PMA does have limitations, including that it does not address valve dysfunction [14]; the procedure could lead to further septic embolization from dislodged infected material during aspiration [15], and residual vegetation can lead to recurrent bacteremia [16].

Our study aims to describe the real-world outcomes of PWID hospitalized with TV IE at an urban hospital between 2016 and 2025 who were treated either with a PMA procedure or surgical intervention (i.e., TV repair/replacement).

## 2. Materials and Methods

This study was a retrospective cohort of PWID admitted to the University of Maryland Medical Center with IE of the TV who underwent an isolated TV procedure between January 2016 and September 2025. The study was reviewed by the University of Maryland’s Institutional Review Board and deemed exempt from full review.

**Figure 1:**
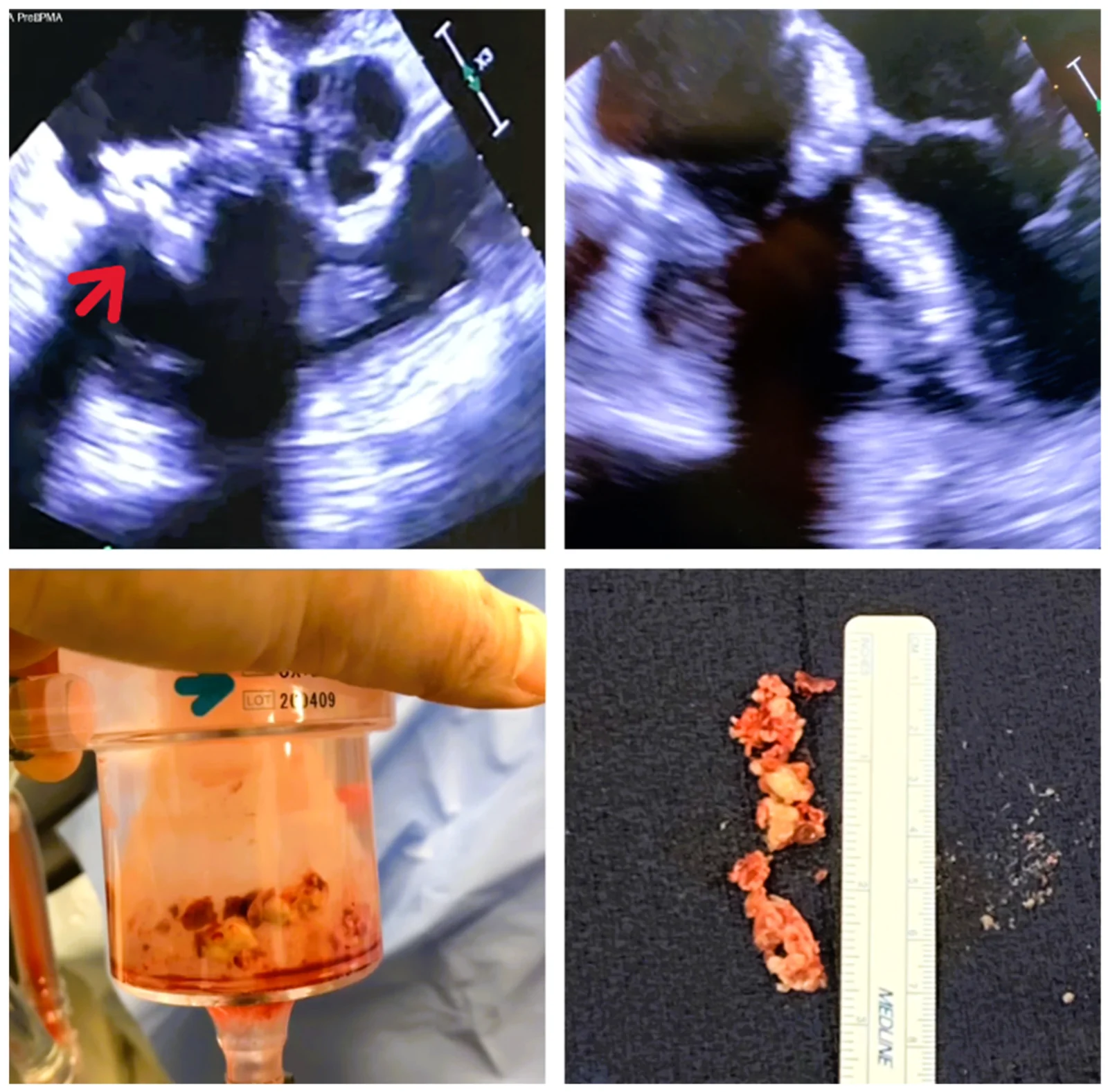
Representative example of vegetation debulking using PMA. Transesophageal echocardiographic mid-esophageal four-chamber view demonstrating a large tricuspid valve vegetation prior to PMA (arrow, (**top left**)) and post-procedural echocardiographic image demonstrating reduction in vegetation burden following aspiration (**top right**). Aspirated material is shown in the filter trap (**bottom left**) and after being flushed out of the trap (**bottom right**).

Patients were identified from the Adult Cardiac Surgery (ACS) registry through the Society of Thoracic Surgeons (STS) database. This list was cross-referenced with an electronic health record data search using diagnostic codes for endocarditis of the TV from the International Classification of Diseases, tenth revision (ICD-10). Patients treated with PMA were further identified using Current Procedural Terminology (CPT) codes for percutaneous thrombectomy, atrial/ventricular thrombus removal, corresponding ICD-10 Procedure Coding System (PCS) codes, and associated Medicare Severity Diagnosis Related Groups (DRG) categories. These patients were matched to ICD-10 diagnosis codes for SUD associated with injection drug use. All PMA procedures in this cohort were performed using the AngioVac system with representative images in Figure 1.

**Figure 2:**
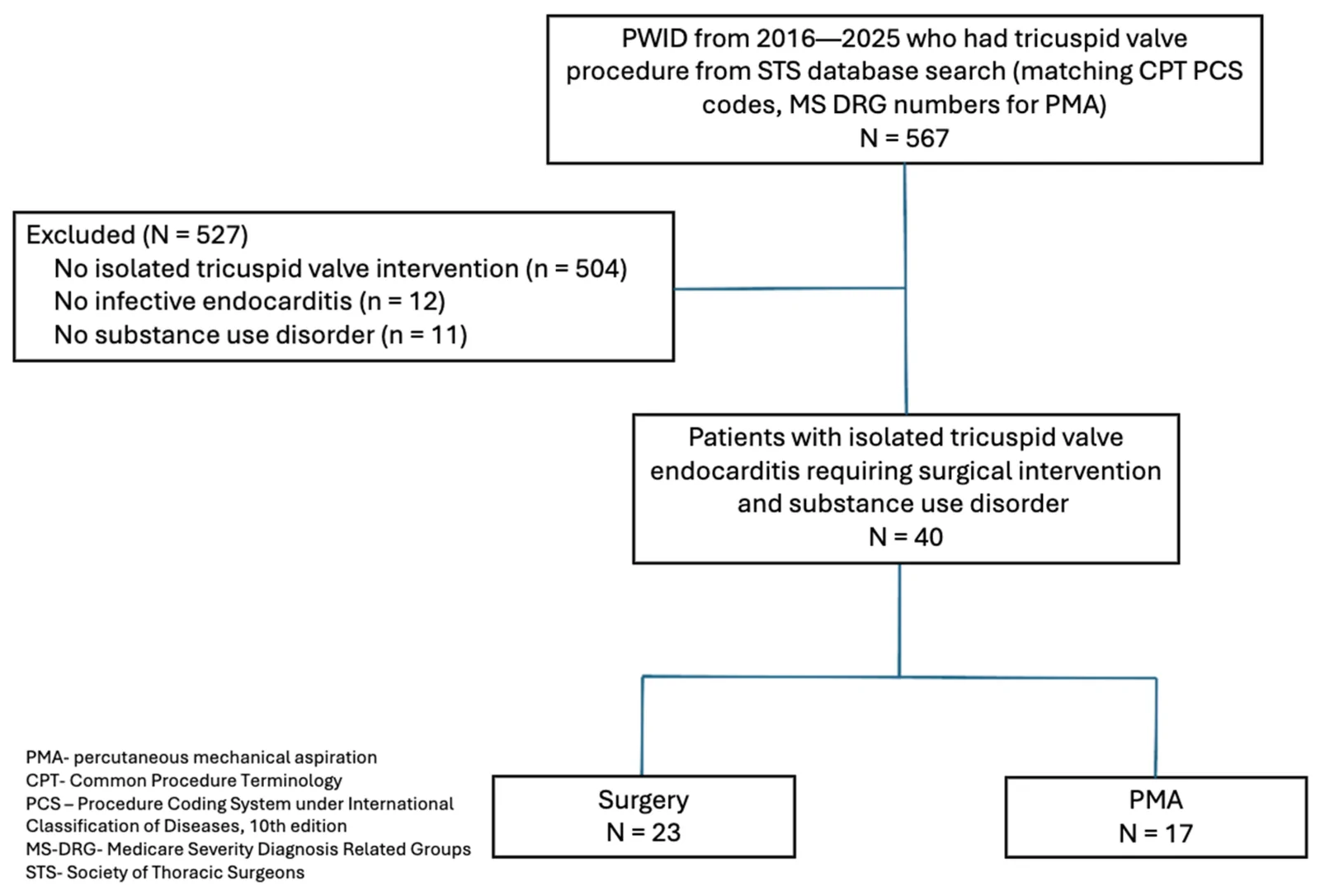
Flow diagram of patient selection for both arms of the study (TVR and PMA).

For a comparison group, we identified patients undergoing isolated TV surgery at our site from the STS ACS database. The list was refined to include only those patients who had a co-morbid SUD diagnosis code and underwent an isolated TV procedure, either tricuspid valve repair or replacement. Patients were excluded if they underwent procedures involving other cardiac valves or any concomitant procedures, such as pacemaker lead insertion, lead extraction, pacemaker placement, procedure for atrial fibrillation, or repair of patent foramen ovale during the same operative encounter. The authors screened both lists for eligibility. Of the 567 patients who underwent TV procedures, 63 underwent isolated TV intervention, of whom 51 had endocarditis and 40 had TV endocarditis related to substance use (see Figure 2 for schema). Of the 40 patients who met inclusion criteria, 17 patients had the PMA procedure, and 23 were managed surgically.

The demographic and clinical characteristics of both groups were examined. Variables were selected based on prior literature, as well as consultation with infectious disease physicians and cardiac surgeons well versed with the procedure.

Information collected from the electronic medical record included demographic characteristics; clinical variables (pathogen type, vegetation size, and severity of tricuspid regurgitation); infection-related variables (duration of bacteremia and prior endocarditis episodes); and treatment-related outcomes (length of antibiotic course and re-admissions due to recurrent infection). Substance use information (substance type and route of use) was aggregated from medical history and urine toxicology results, where available. Vegetation size and tricuspid regurgitation were collected from formal transthoracic and/or transesophageal echocardiography (TEE) reports collected prior to intervention. TEE findings interpreted by experienced echocardiographers during interventions were reviewed by the study team. All but two patients received a substance use consultation during their hospitalization. We reported categorical variables as number (percentage) and continuous variables as mean (standard deviation). To characterize baseline differences between the PMA and TVR groups, the absolute standardized mean difference (SMD) was calculated for each baseline variable. For continuous variables, the SMD was defined as the difference in group means divided by the pooled standard deviation; for categorical variables, it was defined as the standardized difference in proportions. SMD values were reported to descriptively quantify the magnitude of baseline difference between groups.

Given the nonrandomized treatment selection and clinical heterogeneity between groups, outcome comparisons were conducted as descriptive analyses. The primary outcome of the study was a composite clinical success, defined as absence of procedural or post-procedural complications (i.e., access site complications, bleeding, postoperative infection, pericardial effusion, dissection, worsening sepsis, sustained arrhythmias, embolic phenomena, and periprocedural mortality), absence of in-hospital mortality, clearance of bacteremia post-procedure without persistence or recurrence, no requirement for subsequent surgical intervention after procedure, and completion of prescribed antibiotic course. Procedural success was defined as >50% reduction in vegetation size and absence of procedural complications. We compared the PMA and TVR groups based on the proportion achieving these success benchmarks.

Secondary outcomes included procedural success analyzed independently, reintervention within one year (i.e., repeat valve surgery), mortality within one year, readmission within 30 days and one year, need for permanent pacemaker during or after index procedure, median length of hospital stay, need for intensive care unit (ICU) care >48 h (beyond routine post-cardiac procedure monitoring), post-operative vasopressor use lasting >48 h, and post-operative transfusion requirements beyond 48 h (i.e., ongoing hemorrhage requiring treatment).

The categorical outcomes were compared using Fisher’s exact test due to small sample sizes. Odds ratios [OR] with 95% confidence intervals [CI] were calculated for most binary outcomes. The need for transfusion >48 h postoperatively was omitted due to the low prevalence in the dataset. Length of stay (LOS) between groups was compared using the Wilcoxon rank-sum test due to non-normal distribution. Exploratory univariate analyses of the PMA cohort were performed to assess associations between select clinical and procedural factors and clinical success, again using Fisher’s exact test. All statistical tests were two-sided, with a significance threshold of *p* < 0.05.

## 3. Results

### 3.1. Demographic Characteristics

Baseline demographic and clinical characteristics are shown in Table 1, with substantial clinical heterogeneity observed between the two groups. The mean age of the cohort was 34 ± 7 years; 27 patients (67.5%) were female, and six (15%) were Black/African American. Most patients (n = 32, 80%) had documented polysubstance use, while seven patients (17.5%) had isolated opioid use disorder (OUD).

*Staphylococcus aureus* was the most common causative pathogen, identified in 31 (77.5%) cases. In the PMA cohort, one patient had a fungal infection, one had a polymicrobial infection with *Streptococcus dysgalactiae* and *Arcanobacterium haemolyticum*, and two had polymicrobial infections involving *S. aureus* and additional gram-negative pathogens. In the surgical cohort, two patients had fungal infections, and two had polymicrobial/bacterial-fungal infections. Eleven patients (27.5%) had a prior endocarditis episode, and six (15.0%) had undergone prior valve surgery. Twenty-three patients (57.5%) were hepatitis C antibody positive. Thirteen patients (32.5%) had a coexisting mental health diagnosis apart from SUD. Seventeen patients (42.5%) were not on medication for OUD on admission. Thirty-one patients (77.5%) were discharged on medications for OUD.

Echocardiographic findings in both groups included large mobile vegetations ≥2 cm (70.6% PMA vs. 43.5% TVR), severe tricuspid regurgitation (35.3% PMA vs. 60.9% TVR), and right-sided chamber remodeling with RV dilation (52.9% PMA vs. approximately 73.9% TVR). Structural valve destruction including leaflet perforation, flail leaflets, or ruptured chordae, was more frequently observed in the TVR cohort (56.5% vs. 29.4%). Mean duration of bacteremia was shorter in the TVR group (5.7 vs. 6.4 days), while time from bacteremia onset to intervention was longer (17.6 vs. 11.8 days). In the PMA group, 16 (94.1%) patients had pulmonary septic emboli and one (5.9%) had pulmonary and systemic septic emboli. In the TVR group, 15 (65.2%) patients had only pulmonary septic emboli; three (13%) had pulmonary and systemic septic emboli, and five (21.7%) patients had no septic emboli during their admission. Documented reasons PMA patients were deemed poor surgical candidates included high operative risk (n = 10), comorbid substance use (n = 5), team preference (n = 1), and hemodynamic instability (n = 1).

Discharge disposition was varied, and most PMA patients were discharged to skilled nursing facilities (n = 12), though some went home (n = 4) with a plan to complete antibiotic therapy with long-acting lipoglycopeptide therapy (n = 2). For surgical patients, the vast majority were discharged to skilled nursing facilities (n = 18). One person in each group had a patient-directed premature hospital discharge.

### 3.2. Outcomes

Given the baseline heterogeneity between the PMA and TVR groups, clinical outcomes are presented (Table 2) to characterize post-procedural clinical courses without estimating causal treatment effects. The composite clinical success trended numerically higher in the TVR group compared to the PMA group (73.9% vs. 52.9%); however, this difference was not statistically significant (OR 0.40, 95% CI 0.10–1.5; *p* = 0.20). Among PMA patients, failure to achieve clinical success was most commonly due to incomplete systemic antibiotic therapy (n = 5), procedural failure (n = 2), and worsening tricuspid regurgitation requiring valve reintervention within one year along with incomplete completion of systemic antibiotic therapy (n = 1). In the TVR group, clinical failures were due to incomplete antibiotic therapy (n = 4), need for valve reintervention within one year (n = 1), and a procedural complication resulting in worsening heart failure (n = 1).

Procedural success was high in both groups (88.2% PMA vs. 95.7% surgical) and did not differ significantly between groups (OR 0.34, 95% CI 0.03–4.11; *p* = 0.56). Two PMA procedures were classified as procedural failures due to worsening tricuspid regurgitation and <50% reduction in vegetation size, respectively. Two patients from the PMA group and one patient from the TVR group required TV repair within one year (11.8% vs. 4.3%, OR 2.93, 95% CI 0.24–5.33; *p* = 0.56). Two patients each in the PMA group and in the TVR group were deceased at one-year post-index procedure (11.8% vs. 8.7%, OR 1.40, 95% CI 0.14–11.08; *p* = 1.00). While the need for pacemaker placement was uncommon overall, it was slightly higher in the TVR group than in the PMA group (13.0% vs. 5.9%), although not statistically significant (OR 0.42, 95% CI 0.04–4.89; *p* = 0.62).

Median hospital LOS was numerically longer among PMA patients (18 days [IQR 14–36]) compared with surgical patients (16 days [IQR 11–19]), although this difference did not reach statistical significance (*p* = 0.09). Rates of ICU stay exceeding 48 h were similar between groups (35.3% PMA vs. 30.4% surgical; OR 1.25, 95% CI 0.33–4.73; *p* = 1.00). No PMA patients required post-operative blood transfusion beyond 48 h, whereas one surgical patient did (4.3%). Prolonged post-operative vasopressor use (>48 h) was more frequent in the PMA group, although not significantly (29.4% vs. 8.7%; OR 4.38, 95% CI 0.73–26.12; *p* = 0.11). Thirty-day and one-year readmission rates were numerically higher in the TVR group (34.8% and 65.2%, respectively) compared with the PMA group (23.5% and 47.1%), though neither comparison reached statistical significance (30-day: OR 0.58, 95% CI 0.14–2.37, *p* = 0.50; one-year: OR 0.47, 95% CI 0.13–1.71, *p* = 0.34).

### 3.3. PMA Group Analysis

Within the PMA cohort, two patients had a prior episode of endocarditis, and two had prior valve surgery. Baseline tricuspid regurgitation severity was mild in three patients, moderate in four, and severe in ten. Vegetation sizes ranged from 1 × 1 cm to 4 × 1 cm. Of the two PMA patients who died within one year, one had fungal endocarditis and the other had MRSA endocarditis. Regarding antimicrobial management, two PMA patients received long-acting lipoglycopeptides (LA-LGPs); four were transitioned to oral antibiotics to complete therapy, and the remaining 11 completed treatment with intravenous (IV) antibiotics. One PMA patient required permanent pacemaker placement following the intervention.

To better understand outcomes within the PMA cohort, exploratory univariate analyses were performed to identify factors associated with clinical success (Table 3). Vegetation size, severity of tricuspid regurgitation, presence of *S. aureus*, and duration of bacteremia prior to intervention were the selected predictors. Persistence of bacteremia could not be analyzed, as all patients achieved clearance of bacteremia within 48 h of the procedure. None of the evaluated variables were significantly associated with clinical success. Mild/moderate tricuspid regurgitation and shorter duration of bacteremia prior to intervention (<7 days) appeared numerically associated with higher rates of clinical success but were not statistically significant, likely reflecting the small sample size of our PMA cohort.

## 4. Discussion

In this retrospective single-center cohort study, the composite clinical or procedural success of PMA compared to TVR for isolated TV IE in PWID did not show statistically significant differences. Secondary outcomes suggested mixed peri-procedural patterns rather than a uniform advantage for either strategy. ICU utilization was similar between groups, but a higher proportion of PMA patients required vasopressor support for >48 h. This could be partly explained by the fact that some of the patients who underwent surgical repair had returned after completing outpatient IV antibiotic therapy, thereby selecting for healthier individuals. In contrast, patients in the PMA group may have been offered the procedure during the index admission because they were too ill for surgical intervention and there was potential for deterioration prior to discharge. This is reflected in the shorter time from bacteremia onset to intervention in the PMA group compared to the TVR group (11.8 versus 17.6 days). Notably, patients who underwent PMA had numerically lower rates of 30-day and one-year hospital readmission. Most readmissions in the surgical cohort appeared unplanned and were commonly related to recurrent infection or other acute medical complications, consistent with prior literature describing high readmission burden among PWID with infective endocarditis [2,17].

Procedural success rates in the PMA group were comparable to prior reports. In a systematic review of 44 studies including 301 patients, PMA achieved vegetation reduction in 89.2% of cases [10]; our observed rate of procedural success (88.2%) is consistent with these findings. However, clinical success in our PMA cohort was lower than previously reported (52.9% vs. 79.1%), likely reflecting our more stringent composite definition that included completion of antibiotic therapy, absence of complications, and freedom from reintervention. Prior comparative studies evaluating PMA, medical management, and surgery have reported no differences in one-year mortality across treatment strategies [13]. In our cohort, the absolute number of deaths at one year was identical between groups, though the proportion was higher in the PMA group due to a smaller denominator. Given the limited sample size, baseline heterogeneity between groups, and potential institutional variations in practice, the observed differences cannot be attributed to treatment strategy alone. Although PMA may offer peri-procedural advantages in select patients, these benefits are not consistently associated with shorter hospital stays or improved short- or long-term clinical outcomes.

It remains unclear whether the observed differences in clinical outcomes reflect procedure-specific effects or are driven by baseline differences related to patient selection for PMA versus surgery. Given the heterogeneity of clinical indications for PMA and the concern for confounding by indication, exploratory analyses were limited to the PMA cohort. None of the factors considered, vegetation size, tricuspid regurgitation severity, *S. aureus* infection, and prolonged bacteremia, were associated with clinical success, although these analyses were limited by small sample size and should be interpreted cautiously. These findings suggest that disease severity alone does not fully explain outcome variability once PMA is pursued.

Although statistical associations were limited by small sample size, failures to achieve clinical success among PMA patients were often driven by downstream factors, including incomplete antibiotic therapy and progression of valvular dysfunction. This observation supports the concept that PMA serves primarily as a debulking and source control strategy, with durable clinical success dependent on completion of antimicrobial therapy and effective longitudinal care. Inclusion of antibiotic completion within the composite endpoint may therefore reflect social and behavioral factors influencing treatment adherence and longitudinal care engagement rather than procedural efficacy alone.

The use of nontraditional antimicrobial strategies, including LA-LGPs and oral antibiotics, in the PMA cohort likely reflects challenges in arranging outpatient parenteral antimicrobial therapy in PWID [16]. Clinician hesitancy to discharge PWID on IV antibiotics, driven by concerns regarding active substance use, loss to follow-up, and underlying psychosocial issues, has been well documented [18]. While such approaches may be perceived as salvage strategies for patients who decline surgery or are deemed poor surgical candidates [19,20], our institutional experience suggests that PMA, when paired with alternative antimicrobial strategies, may represent a viable option for primary intervention within a patient-centered, shared decision-making framework.

Larger implementation studies evaluating combined nontraditional strategies (e.g., PMA plus oral or LA-LGP antibiotic therapy) are needed, particularly as such approaches may better align with patient priorities around substance use recovery [21]. LA-LGPs represent an additional strategy to improve antimicrobial completion and longitudinal treatment adherence in select PWID with IE [22]. The foundation of right-sided IE management remains prolonged antimicrobial therapy, with surgical intervention considered for patients with persistent bacteremia despite appropriate antimicrobial therapy, recurrent septic pulmonary emboli, severe tricuspid regurgitation causing right-sided heart failure, prosthetic valve involvement, or advanced structural valve destruction [14,15]. Formal evidence-based indications for PMA are not well-established, and selection is individualized and guided primarily by overall operative candidacy and clinical context rather than vegetation size or imaging characteristics. Although large vegetations were common in both cohorts, severe tricuspid regurgitation, advanced structural valve destruction, and more extensive right-sided chamber remodeling were more frequently observed in the TVR cohort. Given that PMA may not correct tricuspid regurgitation, the presence of severe tricuspid regurgitation with right-sided heart failure may favor surgical management.

Both treatment strategies carry important limitations and risks that warrant consideration. Incomplete source control, persistent vegetations, worsening tricuspid regurgitation, and need for surgical reintervention may occur following PMA, particularly given its role primarily as a debulking rather than definitive structural intervention. Procedural risks, including vascular injury, access-site complications, cardiac perforation, distal embolization, and worsening sepsis, have also been reported with PMA, particularly in patients with baseline hemodynamic instability or intracardiac shunting [15]. Conversely, surgical intervention carries risks of perioperative morbidity, recurrent infection, prosthetic valve complications, and reinfection, particularly among PWID with ongoing substance use and complex longitudinal care needs.

This study has several strengths. Our use of multiple data sources increased the likelihood of capturing all relevant isolated TV IE cases. We developed a comparable and more homogeneous study population by excluding cases that did not have confirmed SUD, patients who had multivalve endocarditis, and patients with surgical management outside of the time period during which the PMA cases were completed. The retrospective design was well suited to evaluating an emerging intervention with limited real-world uptake and enabled comprehensive assessment of clinically meaningful composite and secondary outcomes, including one-year reintervention and mortality. Additionally, almost everyone in this cohort was seen by addiction medicine and 78% of patients were discharged on medications for OUD (MOUD), which represents a 35% increase in new initiations of MOUD. Both of these interventions are clearly associated with improved mortality in PWID with endocarditis [14].

Despite the study’s strengths, we acknowledge several important limitations. Due to the retrospective nature of this study and inconsistent documentation, detailed indications for procedural selection could not be ascertained. Importantly, indications for PMA may differ from those for TVR. While surgical decisions are guided by vegetation size and severity of tricuspid regurgitation, PMA is frequently performed with the primary goal of source control rather than definitive repair. In this context, clinical indications such as persistent bacteremia or failure of medical therapy may supersede conventional thresholds (e.g., vegetation size < 2 cm). We were also unable to ascertain how many patients were declined for either procedure, whether patients refused a procedure, and we did not include a comparator group looking at isolated medical management.

There was no uniform treatment algorithm and decisions were based on individual surgeons in consultation with the primary medicine and infectious disease teams. Thus, the potential for provider bias, due to factors such as procedural experience, availability of operative facilities, or concerns regarding comorbidities, in directing one procedure versus another, is not measured or excluded. Treatment selection was individualized and may have been influenced by institutional practice patterns, procedural expertise, and provider preference. Therefore, the differences in baseline risk profiles between the PMA and surgical group may be subject to selection bias, potentially confounding the reported outcomes. Previous studies have reported how clinical severity and social factors influence treatment selection and thus clinical outcomes [23]. Additionally, “high operative risk” was determined from clinical documentation rather than formal prospective risk stratification. Similarly, while a difference in time from bacteremia onset to intervention was observed, 6.4 days for PMA vs. 5.7 days for surgical intervention, the factors driving these delays could not be systematically evaluated or adjusted for.

We did not separate or compare the various TV surgical approaches due to the small sample size, though evidence seems to suggest that valve repair may be associated with lower risk of pacemaker need or recurrent IE [4]. No patients in our cohort underwent tricuspid valvectomy, which has demonstrated worse clinical outcomes [24]. Finally, the relatively small sample size of our study limits the statistical power of formal comparisons between groups. Accordingly, these findings should be interpreted as descriptive and hypothesis-generating, and further study in larger cohorts will be needed before clinical recommendations can be made.

Surgeon perspectives on addiction-related IE further contextualize these findings. Prior surveys indicate that substance use influences operative decision-making, including concerns regarding anticoagulation adherence, valve selection, and technical complexity (e.g., type of valve selected and scar tissue) [25]. Although interdisciplinary case conferences have been shown to facilitate shared understanding and improve care coordination, such a formalized team was not in place at our institution during the study period. Future qualitative and mixed-methods research may better elucidate how early interdisciplinary discussions shape treatment selection and outcomes for PWID with IE.

## 5. Conclusions

This study evaluated outcomes for PWID with isolated TV IE who underwent either valve surgery or PMA. We found no difference between the composite procedural and clinical success metrics in the PMA procedure group compared with the TVR group. Importantly, this study provided clinical context, including antibiotic completion pathways and addiction treatment engagement, rarely captured in larger administrative or registry-based datasets. These findings offer meaningful real-world insight into the management of TV IE in patients with substance use and highlight the importance of care-delivery factors that may influence outcomes beyond the procedural approach alone. In selected patients who are not candidates for immediate surgery, PMA may represent a potential option for achieving source control or as part of a bridge strategy while longer-term management decisions are made, including stabilization of infection or engagement in SUD recovery. Further research in larger prospective cohorts is needed to better define optimal patient selection for PMA versus TVR for isolated TV IE.

## Figures and Tables

**Table 1:** Baseline demographic and clinical characteristics of PWID with TV IE, stratified by PMA versus TVR.

	Overall (n = 40)	PMA (n = 17)	TVR (n = 23)	Absolute SMD ^2^
**Age, years, mean** (**SD**)	34 (7)	36 (7)	32 (6)	0.584
**Gender, N %**				
Female	27 (67.5%)	14 (82.4%)	13 (56.5%)	0.584
**Race, N %**				
White	30 (75%)	12 (70.6%)	18 (78.3%)	0.177
Black or African American	6 (15%)	4 (23.5%)	2 (8.7%)	0.412
Other	4 (10%)	1 (5.9%)	3 (13.0%)	0.247
**Substance use, N %**				
Opioids only	7 (17.5%)	3 (17.6%)	4 (17.4%)	0.007
Polysubstance ^1^	32 (80.0%)	13 (76.5%)	19 (82.6%)	0.153
**Hepatitis C antibody positive, N %**	23 (57.5%)	13 (76.5%)	10 (43.5%)	0.715
**Mental health comorbidity, N %**	13 (32.5%)	4 (23.5%)	9 (39.1%)	0.341
***Staphylococcus aureus*** **infection, N %**	31 (77.5%)	15 (88.2%)	16 (69.6%)	0.470
**Prior Endocarditis, N %**	11 (27.5%)	2 (11.8%)	9 (39.1%)	0.662
**Prior Valve Surgery, N %**	6 (15.0%)	2 (11.8%)	4 (17.4%)	0.160
**Vegetation Size, N %**				
<2 cm	18 (45.0%)	5 (29.4%)	13 (56.5%)	0.569
≥2 cm	22 (55.0%)	12 (70.6%)	10 (43.5%)	0.569
**Duration of bacteremia, days, mean** (**SD**)	6.0 (5.0)	6.4 (4.2)	5.7 (4.2)	0.132
**Time from bacteremia onset to intervention, days, mean** (**SD**)	15.0 (12.0)	11.8 (12.0)	17.6 (11.6)	0.487
**Tricuspid regurgitation, N %**				
Mild	7 (17.5%)	3 (17.6%)	4 (17.4%)	0.007
Moderate	13 (32.5%)	8 (47.1%)	5 (21.7%)	0.553
Severe	20 (50.0%)	6 (35.2%)	14 (60.9%)	0.530
**Structural Valve Damage**	16 (40.0%)	5 (29.4%)	11 (56.5%)	0.385
**Septic Emboli, N %**	35 (87.5%)	17 (100%)	18 (78.3%)	0.745
Pulmonary	31 (77.5%)	16 (94.1%)	15 (65.2%)	0.769
Systemic	4 (10%)	1 (5.9%)	3 (13.0%)	0.247

^1^ Polysubstance use refers to OUD plus the use of at least one additional substance, including cannabis, benzodiazepines, or amphetamines. ^2^ Absolute SMD is reported descriptively to semi-quantify the magnitude of baseline difference between groups. SMD values were not used to assess balance. TV: Tricuspid valve, IE: Infective Endocarditis, PMA: Percutaneous mechanical aspiration, TVR: Tricuspid valve repair/replacement, SMD: Standardized mean difference, SD: Standard deviation, PWID: People who inject drugs, OUD: Opioid use disorder.

**Table 2:** Procedural, clinical, and one-year outcomes among PWID who underwent PMA vs. TVR for TV IE.

	Overall N = 40	PMA N = 17	TVR N = 23	OR (PMA vs. TVR) [95% CI]	*p*-Value
Composite clinical success (absence of complications, survival to discharge, no persistent or recurrent bacteremia post-procedure, no need for surgical re-intervention, and completion of antibiotic course)	26 (65%)	9 (52.9%)	17 (73.9%)	0.40 [0.10–1.50]	0.20
Procedural Success (>50% reduction in vegetation size and absence of complications)	37 (92.5%)	15 (88.2%)	22 (95.7%)	0.34 [0.03–4.11]	0.56
Reintervention on valve within 1 yr, N %	3 (7.5%)	2 (11.8%)	1 (4.3%)	2.93 [0.24–5.33]	0.56
Mortality within 1 year, N %	4 (10.0%)	2 (11.8%)	2 (8.7%)	1.40 [0.14–11.08]	1.00
Readmission within 30 days, N %	12 (30.0%)	4 (23.5%)	8 (34.8%)	0.58 [0.14–2.37]	0.50
Readmission within 1 year, N %	23 (57.5%)	8 (47.1%)	15 (65.2%)	0.47 [0.13–1.71]	0.34
Need for permanent pacemaker, N %	4 (10.0%)	1 (5.9%)	3 (13.0%)	0.42 [0.04–4.89]	0.62
Length of stay, median (IQR)	17 (12–23)	18 (14–36)	16 (11–19)		0.09
Need for ICU admission lasting > 48 h	13 (32.5%)	6 (35.3%)	7 (30.4%)	1.25 [0.33–4.73]	1.00
Need for post-operative vasopressor therapy > 48 h postoperatively	7 (17.5%)	5 (29.4%)	2 (8.7%)	4.38 [0.73–26.12]	0.11
Need for transfusion > 48 h postoperatively	1 (2.5%)	0 (0.0%)	1 (4.3%)		

TV: Tricuspid valve, IE: Infective Endocarditis, PMA: Percutaneous mechanical aspiration, TVR: Tricuspid valve repair/replacement, SMD: Standardized mean difference, SD: Standard deviation, OR: Odds ratio, CI: Confidence Interval, IQR: Interquartile Range, PWID: People who inject drugs, ICU: Intensive care unit.

**Table 3:** Exploratory univariate analysis of factors associated with clinical success among patients who underwent PMA.

	Clinical Success n/N (%)	Clinical Failure n/N (%)	Fisher’s Exact *p*-Value
Vegetation Size, ≥2 cm	6/12 (50%)	6/12 (50%)	1.00
Vegetation Size, <2 cm	3/5 (60.0%)	2/5 (40.0%)	-
Severe tricuspid regurgitation	5/10 (50.0%)	5/10 (50.0%)	0.62
Mild/Moderate tricuspid regurgitation	5/7 (71.4%)	2/7 (28.6%)	-
*Staphylococcus aureus* infection	8/15 (53.3%)	7/15 (46.6%)	1.00
Other pathogen infection	1/2 (50%)	1/2 (50%)	-
Bacteremia ≥ 7 days prior to intervention	2/5 (40%)	3/5 (60%)	0.62
Bacteremia < 7 days prior to intervention	7/12 (58.3%)	5/12 (41.7%)	

PMA: Percutaneous mechanical aspiration.

## Data Availability

The raw data supporting the conclusions of this article will be made available by the authors on request.

## Abbreviations

The following abbreviations are used in this manuscript:

ACS	Adult cardiac surgery
CI	Confidence interval
CPT	Current procedural terminology
DRG	Diagnosis related groups
ICD-10	International classification of disease, tenth revision
ICU	Intensive care unit
IE	Infective endocarditis
IQR	Interquartile range
IV	Intravenous
IVDU	Intravenous drug users
LA-LGP	Long-acting lipoglycopeptide
LOS	Length of stay
MOUD	Medications for opioid use disorder
MRSA	Methicillin resistant *Staphylococcus aureus*
OR	Odds ratio
OUD	Opioid use disorder
PCS	Procedure coding system
PMA	Percutaneous mechanical aspiration
PWID	People who inject drugs
SMD	Standardized mean difference
STS	Society of Thoracic Surgery
SUD	Substance use disorder
TEE	Transesophageal echocardiography
TV	Tricuspid valve
TVR	Tricuspid valve repair/replacement
